# Group Analysis of *Q* Values Calculated with Tangential Radius of Curvature from Human Anterior Corneal Surface

**DOI:** 10.1155/2018/7263564

**Published:** 2018-04-17

**Authors:** Zheren Xia, Chengmin Lin, Xueping Huang, Jinglu Ying, Mingguang Shi, Suilian Zheng

**Affiliations:** ^1^Department of Ophthalmology, The Second Affiliated Hospital and Yuying Children's Hospital of Wenzhou Medical University, Wenzhou 325027, China; ^2^Department of Ophthalmology, Wenzhou Hospital of Integrated Traditional Chinese and Western Medicine, Wenzhou 325000, China; ^3^Department of Information and Engineering, Wenzhou Medical University, Wenzhou 325035, China; ^4^Department of Ophthalmology, Sir Run Run Shaw Hospital, School of Medicine, Zhejiang University, Hangzhou 310016, China

## Abstract

**Objective:**

To calculate the *Q* values from the human anterior corneal surface with the tangential radius of curvature and analyze its distribution characteristics in different age and refractive status groups.

**Methods:**

Tangential power maps of the anterior cornea from Orbscan II were acquired for 201 subjects' right eyes. They were divided into groups of adults and children and then divided further into subgroups according to the refraction status. The *Q* values of each semimeridian were calculated by the tangential radius with a linear regression equation. The *Q* value distribution in both the nasal cornea and temporal cornea were analyzed.

**Results:**

The mean temporal *Q* values of the emmetropia group of adults and all children's groups were significantly different from the mean nasal *Q* value. The mean nasal corneal *Q* values were more negative in children. The adult group showed differences only in the low myopia group. The mean *Q* value of the nasal cornea among different refractive groups of children was significantly different, and so was the temporal cornea between the adult myopia and emmetropia group.

**Conclusion:**

The method using the tangential radius of curvature combined with linear regression to obtain anterior surface *Q* values for both adults and children was stable and reliable. When we analyzed the anterior corneal *Q* value, area division was necessary.

## 1. Introduction

The anterior surface of the cornea is not perfectly spherical, but aspherical. Several mathematical models [1, 2] have been proposed to describe the anterior corneal shape. Baker proposed a quadratic curve theory in 1943: *y*^2^ = 2*r*_0_*x* − *px*^2^ (*r*_0_ is the vertex radius and *p* is a shape factor). The anterior surface of the corneal cross section can be represented as a parabola, circle, ellipse, and hyperbola. However, this research has never stopped; in 1968, Mandell proposed an elliptical cross-sectional model formula characterizing the human cornea: (*x*^2^/*a*^2^) + (*y*^2^/*b*^2^) = 1. Most previous studies have found that the anterior corneal surface is prolate [3, 4]. In 1991, Bennett and Rabbetts [5] derived the anterior surface of the cornea cross section with the calculation formula, *r*_*s*_^2^ = *r*_0_^2^ + (1 − *p*)*y*^2^, to calculate the asphericity by the sagittal radius of curvature (*r*_*s*_) using a keratometer. Although Bennett's equation is widely used in the studies of corneal shape, it still has its limitations. Because it is based on the assumption of the paraxial optical theory, it can only approximately depict the corneal shape in the central region and only provide a mean *Q* value or two main meridian *Q* values of the anterior surface of the cornea.

**Figure 1:**
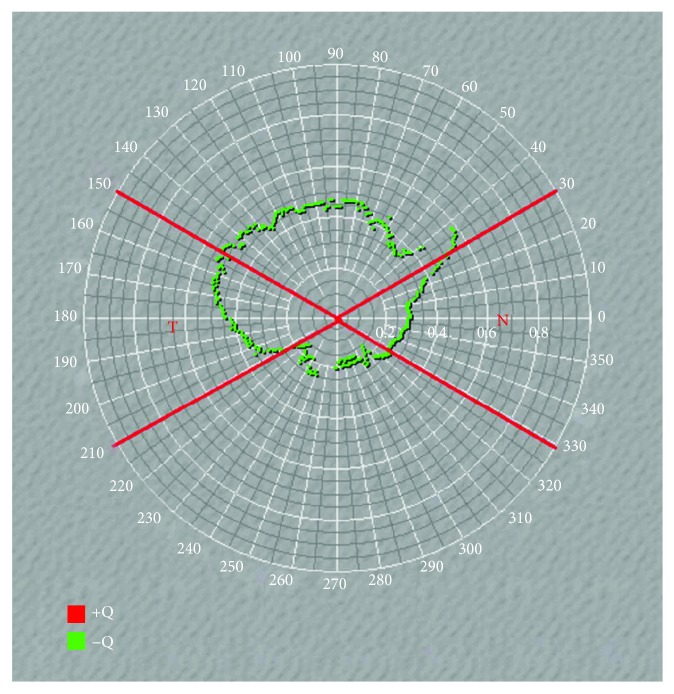
An example of the *Q* value distribution of the right eye; we selected the nasal cornea (330°–29°) and the temporal cornea (150°–209°) in our study.

Corneal topography is commonly presented as an axial (sagittal) power, tangential power, or elevation map. The tangential radius of curvature (*r*_*t*_) is a true radius of curvature that can better represent the corneal shape and local curvature changes. In 2012, Ying et al. [6] proposed a method for calculating the corneal asphericity (*Q*) and analyze the characteristics of the anterior corneal shape using the tangential radius. In 2013, Zheng et al. [7] elucidated the 360-semimeridional variation rule of the *Q* value using the polynomial fitting and constructed a customized 3-D model of the anterior corneal surface. In this study, we calculated the nasal cornea (330°–29°) and the temporal cornea (150°–209°) *Q* values with the tangential radius of curvature (Figure 1) and analyzed the *Q* value distribution characteristics of adults and children with different refractive status.

## 2. Materials and Methods

### 2.1. Subjects

201 Chinese patients who met the inclusion criteria were recruited from January 2014 to November 2015 in the Department of Ophthalmology of the Second Affiliated Hospital of Wenzhou Medical University. Only the right eyes were selected as objects of research. Other than refractive error, all included eyes were free of ocular disease and previous refractive surgery. The subjects were divided into two groups according to age: children: 6–14 years old and adults: 18–35 years old. Each group was then further divided into subgroups according to their refractive status. The adult group includes (1) the very high myopia group: spherical equivalent ≥ −9.00D; (2) high myopia group: −9.00D > spherical equivalent ≥ −6.00D; (3) moderate myopia group: −6.00D > spherical equivalent ≥ −3.00D; and (4) low myopia group: −3.00D > spherical equivalent ≥ −0.50D. The children's group includes (1) hyperopia: +3.00D ≥ spherical equivalent ≥ + 0.75D and (2) myopia: −6.00D > spherical equivalent ≥ −0.50D.

All subjects underwent a full ophthalmic examination including visual acuity, manifest refraction, slit lamp biomicroscopy, fundus examination, and dilated refraction. All subjects had a visual acuity of 6/6 or better. Subjects with corneal astigmatism ≥ 1.0D or irregular astigmatism were excluded.

All subjects gave their informed consent according to the Declaration of Helsinki, and the study was approved by the Second Affiliated Hospital of Wenzhou Medical University Research Ethics Committee, China.

### 2.2. Data Acquisition

All participants had corneal topography examination with the Orbscan II corneal topography system (3.00 E, Bausch & Lomb, Rochester, NY, USA). All examinations were performed by one skilled technician. Three images were obtained from each subject. Topographic images, in which ≥75% of the data was available, were selected for further analysis. The *Q* value was calculated for each of these maps for a given meridian and then averaged.

### 2.3. *Q* Value Calculation

The tangential radius of curvature (*r*_*t*_), the perpendicular distance from the point to the optical axis (*y*) of all data points on a semimeridian, and the vertex radius of curvature (*r*_0_ value) were obtained from the raw data of the tangential power map of the anterior corneal surface. The data points were arranged on a semimeridian at 0.1 mm intervals from the corneal vertex to 3.0 mm away. The interval between two semimeridians was 1°. Our previously published paper introduced the derivation of the equation in detail for the *Q* value calculation by the tangential radius [6]. We have the equation *y*^2^ = *b* + *cr*_*t*_^2/3^, where *b* and *c* were constants. A straight-line graph of *y*^2^ (on the ordinate) versus *r*_*t*_^2/3^ (on the abscissa) was plotted. Using linear regression, we obtained *Q* = −(*b*^2^/*c*^3^). The straight line gives the coefficient of determination (*R*^2^). Considering the reliability of the linear regression equation, the coefficient of determination (*R*^2^) should be >0.5. A computer program (Analysis Software of Three-Dimensional Model for Human Anterior Corneal Surface, V1.0, Chinese patent no. 2012SR007680) was used for all computations.

Due to the influence of the upper and lower eyelids, the coefficient of determination (*R*^2^) in the vertical cornea is not as good as in the horizontal cornea. So in our study, only the horizontal cornea was selected and further divided into the nasal cornea (330°–29°) and the temporal cornea (150°–209°). The mean value of all semimeridian *Q* values in this area was analyzed.

### 2.4. Statistical Analysis

Statistical analyses were performed (SPSS version 15.0), and the differences of the *Q* values between the temporal and nasal cornea were evaluated with a paired *t*-test. The differences of the *Q* values between different groups were analyzed with one-way ANOVA. The *Q* values of the adult and children's groups were compared with results from an independent sample *t*-test. A value of *p* < 0.05 was considered statistically significant.

## 3. Results

### 3.1. Coefficients of Determination

The median values of the coefficient of determination (*R*^2^) of all semimeridians in the two groups were both above 0.9. All coefficients of determination were above 0.5.

### 3.2. The Mean *Q* Value of Each Group in the Temporal and Nasal Cornea

The semimeridian *Q* values of 201 eyes' horizontal cornea (containing nasal and temporal cornea) were between −0.9361 and −0.0348, as obtained with the one-sample Kolmogorov-Smirnov test for normality test, showing normal distribution in each group. The average *Q* values of the nasal and temporal cornea of all eyes were −0.37 ± 0.14 and −0.25 ± 0.11, respectively. In the adult group, the nasal and temporal mean *Q* values were −0.34 ± 0.13 and −0.26 ± 0.12, while in the children's group, they were −0.42 ± 0.15 and −0.23 ± 0.08, respectively. The nasal and temporal corneal *Q* values in both groups were significantly different (*p* < 0.05).

### 3.3. Comparative Analysis of Mean *Q* Values of Different Refractive Groups (Tables 1–3)

In our study, the mean *Q* values of the nasal cornea among different refractive groups of children were significantly different, and so was that of the temporal cornea between the adult emmetropia group and myopia group (*p* < 0.05). Other groups show no significant difference.

### 3.4. Comparative Analysis of Mean *Q* Values of Different Age Groups (Tables 1–3)

In our study, the subjects were divided into adult and children's groups. The difference between the two groups was statistically significant for both the temporal and nasal cornea (nasal: *p* < 0.001; temporal: *p* = 0.032).

## 4. Discussion

Most corneal topography instruments adopt Bennett's sagittal curvature radius formula. The shortcomings and deficiencies of this formula have been discussed in the Introduction. In previous studies, the *Q* values were obtained directly from the corneal topography instruments [3], which was only a mean *Q* value of the whole cornea or two principal corneal meridians. Furthermore, as the shape of the cornea is not a rotational symmetry model, Bennett's sagittal curvature radius formula is not adequate to calculate the *Q* value. As has been reported from China and other countries, the mean *Q* value is within a range of fluctuations from −1.028 to +0.47 [8–11]. One reason for the difference is the use of different instruments such as Placido disk systems and slit scanning or Scheimpflug devices. Another reason is some of the studies calculated over different corneal chord diameters. As shown in the former studies, with increasing diameter, the asphericity becomes more negative [12, 13]. In our study, the semimeridian *Q* values have a normal distribution and range between −0.0348 and −0.9361, 80% of which are between −0.11 and −0.55. Further, the mean *Q* value of all subjects is −0.31 (the *Q* value of each group fluctuates from −0.27 to −0.35). Compared to other studies, our fluctuations in the *Q* values are relatively small. In our study, the anterior surface of the corneal cross section is prolate, which is consistent with most previous results.

In the present study, the temporal *Q* values of the emmetropia group of adults and all children's groups were significantly different from the nasal *Q* values. The nasal corneal *Q* values were more negative (*p* < 0.05). This is consistent with Fuller and Alperin [14] and Zhang et al. [15]. Zhang et al. analyzed 1052 subjects and concluded that the nasal cornea had a more negative *Q* value than the temporal cornea. They interpreted that it may be because the internal rectus muscle located closest to the limbus, or the nasal sclera, has a flatter shape. This may suggest that an asymmetric design is necessary for contact lens fitting.

The relationship between the corneal *Q* value and refractive errors is still uncertain. In this study, adults and children were divided into different groups according to the refractive status. For adults, the difference between the myopia and emmetropia group for temporal corneal *Q* values was statistically significant, where the myopia group has a more negative *Q* value. In contrast, for children, the difference among the different refractive groups was statistically significant only for the nasal corneal *Q* values. It seems that adults and children exhibit no consistent trend for the corneal *Q* value. Zhang et al. [15] insisted that the *Q* value tends to be more negative with the increase in myopia, but they did not analyze the *Q* values of different partitions of the cornea. Some studies [9, 16] had a contrary view that with the increase in myopia, the cornea tends to be more oblate. Nieto-Bona et al. [17] insisted that the *Q* value had no significant relationship with the spherical equivalent (SE), and that in order to define this relationship, one must consider the corneal refractive power and the eye axis length. Fuller and Alperin [14], Mainstone et al. [18], and Atchison [19] also insisted that the *Q* value has no relationship with the refractive status. Fuller and Alperin and some other studies [17, 20] suggested that these differences may relate more to study design and to the geometric properties of the eye. Therefore, the current relationship between the *Q* value and corneal refractive status is still controversial. Instead of analyzing the mean *Q* value as in other studies, we compared different partitions of the cornea. By further division into more partitions, we may be able to find the tendency of the corneal *Q* value. Furthermore, the corneal chord must be accounted for when comparing these studies about the relationship between the refractive error and *Q* values. Most studies [9, 14, 15, 19] measured asphericities at the 6 mm diameter, which were consistent with our study.

In this study, subjects were divided into the adult group (18–35 years) and children's group (6–14 years) and the mean nasal and temporal corneal *Q* values in these two groups were significantly different. Previous studies have usually focused on the mean *Q* value for the entire cornea, and there has been no interpartition comparison. Zhang et al. [15] found that although there are some differences between different age groups, the *Q* value has no correlation with age. Scholz et al. [11] studied 487 subjects from 17 to 81 years and found that the *Q* value has no correlation with age. Atchison [19] arrived at similar conclusions. Davis et al. [21] studied 643 children with 5 years of follow-up and found that with increasing age, the *Q* value becomes more positive. In this study, we analyzed both the nasal and temporal *Q* values and found that the mean nasal *Q* value of the children's group was more negative than the adult group while their mean temporal *Q* value was less negative, which may be related to the asymmetry in the development of the eye. However, as this study was designed to compare the *Q* value with the intercontrast method, there was no equal myopic subjects in two groups, and no hyperopes in the adult group. This may have introduced a systematic bias and calls for further research.

In recent years, different studies [22–25] have shown the importance of the asphericity (*Q* value) of the cornea. Especially for the patients after refractive surgeries, the changes of the *Q* values may increase the spherical aberration and influence their contrast sensitivity [24, 25]. Our study provided both nasal and temporal *Q* value distribution characteristics of Chinese populations and may contribute more information for the refinement of the model of corneal shape to optimize *Q*-customized ablation. Besides, these findings may offer potential applications in intraocular lens calculations, orthokeratology lens, and other contact lens designs.

A limitation of this study is that only the horizontal cornea was selected for analysis. For reasons we interpreted in Materials and Methods, we hope for more reliable data to approve this new method. And we will try to analyze the whole cornea in the future.

In summary, our method that uses tangential radius of curvature combined with linear regression to obtain anterior surface *Q* values for both adults and children was stable and reliable. When we analyzed the anterior corneal *Q* value, area division was necessary, which can result in better clinical applications.

## Figures and Tables

**Table 1:** The *Q* values of the anterior cornea of the adult group (*n* = 123).

	Nasal	Temporal	*n*	*p*
Myopia	−0.33 ± 0.13	−0.30 ± 0.13	79	0.083
Emmetropia	−0.35 ± 0.12	−0.20 ± 0.09	44	<0.001
*p*	0.287	<0.001		

Note: *n* = number of eyes.

**Table 2:** The *Q* values of the anterior cornea of the adult myopia group (*n* = 79).

	Nasal	Temporal	*n*	*p*
Very high myopia	−0.28 ± 0.09	−0.35 ± 0.13	11	0.142
High myopia	−0.32 ± 0.08	−0.31 ± 0.07	22	0.659
Moderate myopia	−0.32 ± 0.14	−0.29 ± 0.13	23	0.435
Low myopia	−0.36 ± 0.16	−0.26 ± 0.15	23	0.005
*p*	0.395	0.235		

Note: *n* = number of eyes.

**Table 3:** The *Q* values of the anterior cornea of the children's group (*n* = 78).

	Nasal	Temporal	*n*	*p*
Myopia	−0.31 ± 0.11	−0.30 ± 0.13	11	0.031
Emmetropia	−0.42 ± 0.14	−0.23 ± 0.08	44	<0.001
Hyperopia	−0.46 ± 0.17	−0.24 ± 0.09	23	<0.001
*p*	0.028	0.919		

Note: *n* = number of eyes.
